# Type I IFNs contribute to upregulation of PD-L1 during *Chlamydia trachomatis* infection

**DOI:** 10.1128/iai.00040-25

**Published:** 2025-03-12

**Authors:** Nicole V. Reinhold-Larsson, Michael N. Starnbach

**Affiliations:** 1 Department of Microbiology, Harvard Medical School 1811 , Boston, Massachusetts, USA; University of Pennsylvania Perelman School of Medicine, Philadelphia, Pennsylvania, USA

**Keywords:** T cells, cytotoxic T cells, bacterial, *Chlamydia*, PD-L1, costimulation

## Abstract

*Chlamydia trachomatis* is an obligate intracellular bacterial pathogen that if left untreated can cause reproductive harm. Failure of natural adaptive immunity results in chronic and repeat infections. In efforts to understand the failure of adaptive immunity, we have previously discovered that CD8^+^ T cells, normally integral for controlling intracellular pathogen infections, are misprogrammed by PD-1/PD-L1 signaling during *in vivo C. trachomatis* infection and fail to mount a protective response. Seeking to uncover the pathways and host factors involved in PD-L1 upregulation that may lead to CD8^+^ T-cell inhibition, we discovered that *C. trachomatis* triggers the secretion of host type I interferons (IFNs) that are necessary and sufficient to upregulate PD-L1 *in vitro*. Additionally, secretion of type I IFNs is dependent on *C. trachomatis* development and its type III secretion system. We have also validated that type I IFNs contribute to upregulation of PD-L1 during *C. trachomatis* infection *in vivo* using a mouse model of infection. Overall, these findings reveal that *C. trachomatis* induction of this host pathway may contribute to adaptive immune evasion.

## INTRODUCTION

*Chlamydia trachomatis* is responsible for the most commonly reported sexually transmitted infection (STI) in the United States (1, 2). *Chlamydia* infection is treatable with antibiotics, but untreated infections can result in disease sequelae such as pelvic inflammatory disease, ectopic pregnancy, and infertility (3, 4). Because asymptomatic cases are common, many infections are left untreated (5). Furthermore, even with antibiotic treatment, previously infected individuals are susceptible to repeat infections (6), suggesting a failure to acquire adaptive immunity following natural infection. Given these challenges, a vaccine would be the most effective method to control this epidemic.

In choosing which elements of adaptive immunity might be most effective against *Chlamydia* if stimulated by a vaccine, we had considered CD8^+^ T cells as a candidate for controlling this intracellular pathogen. CD8^+^ T cells are normally critical for controlling other obligate intracellular pathogens, such as viruses, because they kill infected cells directly and deprive these organisms of their replicative niche (7). However, our previous studies have shown that memory CD8^+^ T-cell formation is impaired following natural *C. trachomatis* infection (8–10). Furthermore, we and others have known for some time that CD8^+^ T cells are dispensable for controlling infection (8, 11, 12). In the absence of a functional CD8^+^ T-cell response, it has become increasingly clear that IFN-γ producing CD4^+^ T cells are actually the major effector of *C. trachomatis* clearance (13, 14). Historically, CD4^+^ T cells are thought of as helper immune cells that act primarily through potent cytokine secretion (15). However, it is now appreciated that CD4^+^ T cells can develop a cytotoxic phenotype similar to classical CD8^+^ T cells and are capable of directly killing infected cells to mediate protection (16–18). Indeed, our group has shown that cytotoxic CD4^+^ T cells contribute to protection from *C. trachomatis* infection (19). Thus, it is clear that direct killing of infected cells by adaptive immunity is critical for protection against *C. trachomatis* to such an extent that CD4^+^ T cells have acquired cytotoxic capabilities to compensate for the defective CD8^+^ T-cell response.

Since direct cytotoxic killing of *C. trachomatis*-infected cells significantly contributes to protection, it is intriguing that CD8^+^ T cells do not contribute to protection. Interestingly, *Chlamydia-*specific CD8 T cells stimulated *in vitro* (20), but not *in vivo* (8), are capable of contributing to protection when transferred into mice. Thus, some conditions of CD8 T-cell priming that occur very early in *C. trachomatis* infection likely contribute to the defective CD8^+^ T-cell response. A previous work has established that upregulation of Programmed Death Ligand-1 (PD-L1), an immunoinhibitory ligand, significantly contributes to defective primary and memory CD8^+^ T-cell responses during *C. trachomatis* infection (8). However, the pathways and host factors involved in driving *C. trachomatis*-induced PD-L1 expression had yet to be explored. Here, we show that *C. trachomatis* development is required to induce PD-L1 upregulation *in vitro* through a Type III secretion system (T3SS)-dependent mechanism. Additionally, we found that *C. trachomatis* infection triggers the production and secretion of type I IFNs that are necessary and sufficient to induce PD-L1 upregulation *in vitro*. Finally, we validated that type I IFNs contribute to upregulation of PD-L1 during *C. trachomatis* infection *in vivo*.

## MATERIALS AND METHODS

### Growth and isolation of bacteria

*Chlamydia trachomatis* serovar L2 (434/Bu; ATCC) was propagated in McCoy cells as described previously (21, 22). Purified elementary bodies were aliquoted and stored at −80°C in SPG buffer (250 mM sucrose, 10 mM sodium phosphate, 5 mM L-glutamic acid) and thawed immediately prior to use.

### Mice

WT (C57Bl/6J) or IFNαR1^−/−^ mice were purchased from The Jackson Laboratory (Bar Harbor, ME). Mouse care was supervised by the Harvard Medical School Center for Animal Resources and Comparative Medicine staff, and all experiments were approved by the Institutional Animal Care and Use Committee at Harvard.

### Isolation of mouse embryonic fibroblasts (MEFs) and cell culture

Briefly, 12.5- to 14.5-day-old WT (C57Bl/6J) or IFNαR1^−/−^ embryos, excluding the head and liver, were isolated, minced, and homogenized for 10 min with 0.05% trypsin and 0.53 mM EDTA (Corning) treatment at 37°C. Cell homogenate solution was diluted with Dulbecco's modified Eagle's medium (Corning 10–017-CV) supplemented with 10% fetal bovine serum (FBS), 1 mM sodium pyruvate (Gibco), and 10 mM HEPES (Gibco) and subsequently filtered (100 µm, falcon). MEFs were spun at 1,500 RPM for 5 min, and the cell pellet was resuspended in the above media. MEF resuspension was transferred to a treated flask(s) according to the number of embryos isolated and incubated at 37°C in a humidified 5% CO_2_ atmosphere. Primary MEFs were frozen and stored in complete growth media supplemented with 30% FBS and 10% dimethyl sulfoxide (DMSO).

### *In vitro C. trachomatis* infections and supernatant transfers

MEFs were plated 24 -48 h prior to infection. MEFs were infected with *C. trachomatis* at a multiplicity of infection (MOI) of 1 or mock infected with SPG and spun at 2,800 RPM for 30 min at 37°C prior to incubation at 37°C in a humidified 5% CO_2_ atmosphere. For C1 (N′-(3,5-dibromo-2-hydroxybenzylidene)-4-nitrobenzohydrazide) (ChemBridge compound ID# 5113023) experiments, the inoculum was replaced with fresh media containing DMSO or C1 at indicated time points. For supernatant transfer experiments, the supernatant from MEFs infected with *C. trachomatis* or mock infected with SPG was collected at 24 hours post infection (hpi) and sterilized using a 0.2-μm filter. Where indicated, supernatants were heated at 56°C for 30 min with agitation every 5 min. The collected supernatants were immediately transferred onto naïve MEFs for heating supernatant experiments. For all other experiments, supernatants were stored at −80°C and thawed immediately prior to supernatant transfers.

### RNA isolation and reverse transcription-quantitative PCR (RT-qPCR)

The total RNA from MEFs was harvested at indicated time points using the RNeasy kit (Qiagen) according to the manufacturer’s protocol. Reverse transcription of total RNA to cDNA (Thermo Fisher) was performed according to the manufacturer’s protocol. Quantitative PCR was performed on 1 ng of cDNA using PowerUp SYBR Green Master Mix for qPCR (Applied Biosystems) on the Applied Biosystems QuantStudio 5 PCR machine. Transcript levels were normalized to GAPDH. The following primer sequences were used: *gapdh*, F, 5′-GGTGCTGAGTATGTCGTGGA-3′, R, 5′-CGGAGATGATGACCCTTTTG-3′; *pdl1*, F, 5′-TGGACAAACAGTGACCACCAA-3′, R, 5′-CCCCTCTGTCCGGGAAGT-3′

### Flow cytometry

Single-cell suspensions were immediately stained with fluorescently conjugated antibodies against cell surface markers. For *in vitro* MEF experiments, MEFs were stained with the following from Biolegend: anti-PD-L1 PE (clone 10F.9G2) and Zombie NIR viability dye. For *in vivo* mouse experiments, cells were stained with the following from Biolegend unless denoted otherwise: anti-CD326 PE-Cy7 (clone G8.8), anti-I-Ab FITC (clone AF6-120.1), anti-Cd11c APC-Cy7 (clone N418), anti-PD-L1 PE (clone 10F.9G2), and LIVE/DEAD Fixable Aqua Dead Cell Dye (Invitrogen). An LSR II flow cytometer (BD Biosciences) was used to collect data, and FlowJo (Tree Star, Ashland, OR) was used for data analysis.

### Enzyme-linked immunosorbent assay (ELISA)

Filter-sterilized supernatants from MEFs or homogenized mouse upper genital tracts were frozen at −80°C and thawed immediately prior to use. Mouse IFN-α ELISA (Mabtech) and Duoset IFN-β ELISA (R&D Systems) were performed according to the manufacturer’s protocol for *in vitro* MEF supernatant experiments. Mouse IFN-α All Subtype ELISA kit, high sensitivity (PBL Assay Science), and Mouse IFN-β ELISA kit, high sensitivity (PBL Assay Science), were used for IFN detection in mouse uterus homogenates according to the manufacturer’s protocol.

### Recombinant IFN-α and IFN-β

Naïve MEFs were incubated with active recombinant mouse IFN-α (Abcam) or IFN-β (R&D Systems) where indicated for 6 h prior to RT-qPCR or flow cytometry analysis.

### Neutralizing antibodies

Thawed filter-sterilized supernatants from infected MEFs were incubated with rat IgG1 Isotype control (R&D Systems), rabbit IgG control (R&D Systems), anti-IFN-α (PBL Assay Science), or anti-IFN-β (R&D Systems) for 1 h prior to transfer onto naïve MEFs. Six hours post-transfer, MEFs were analyzed by RT-qPCR and flow cytometry.

### Infection of mice and preparation of tissue

Six- to 8-week-old mice were subcutaneously treated with 2.5 mg of medroxyprogesterone 7 days prior to infection. Mice were transcervically infected with 5 × 10^6^ inclusion forming units (IFUs) of *C. trachomatis* or mock infected with SPG using a Non-Surgical Embryo Transfer (NSET) device (ParaTechs, Lexington, KY) as described previously (13). At 24 hpi, the upper genital tracts and uterine draining lymph nodes were harvested. Single-cell suspensions of upper genital tracts were obtained using a multi-tissue dissociation kit 1 (Miltenyi Biotec) according to the manufacturer’s protocol. Single-cell suspensions of uterine draining lymph nodes were obtained by grinding between frosted microscope slides.

### Detection of *C. trachomatis* by quantitative PCR (qPCR)

*C. trachomatis* burden was quantified using qPCR as previously described (23). Briefly, DNA was extracted from mouse upper genital tract homogenate using the DNeasy blood and tissue kit (Qiagen) according to the manufacturer’s protocol. DNA was analyzed using *C. trachomatis* 16S (F, 5′-GGAGGCTGCAGTCGAGAATCT-3′; R′, 5′-TTACAACCCTAGAGCCTTCATCACA-3′: IDT, San Jose, CA) and mouse GAPDH (Applied Biosystems) primer pairs and dual-labeled probes.

### Statistical analysis

Prism software (GraphPad) was used for all statistical analysis. Differences were considered statistically significant if the *P* value was less than 0.05 (*P < 0.05, **P < 0.01, ***P < 0.01, ***P < 0.001, ****P < 0.0001). All data are represented as means ± standard error of the mean (SEM).

## RESULTS

### Upregulation of PD-L1 is dependent on *C. trachomatis* development and the T3SS

**Fig 1:**
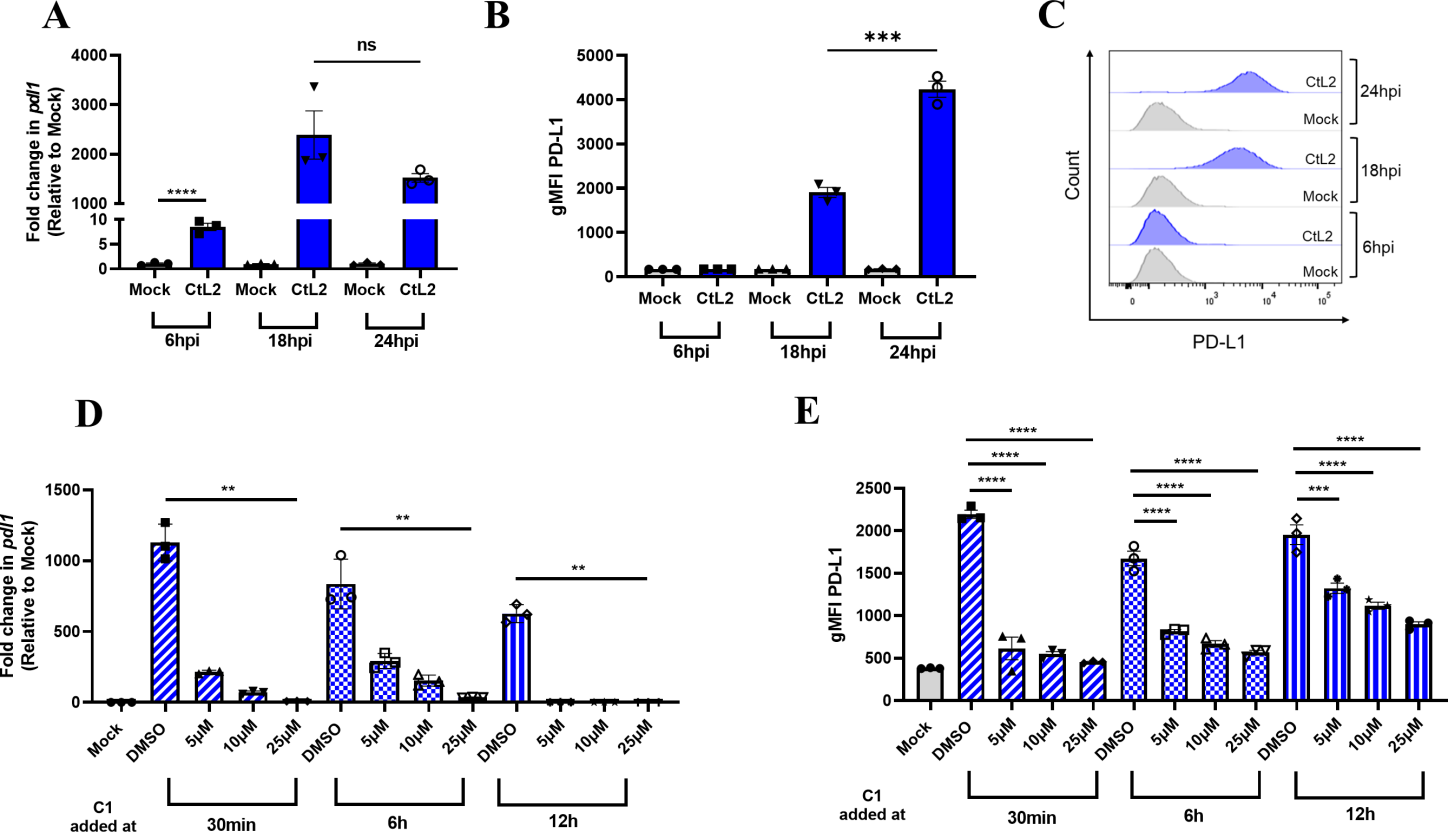
*C. trachomatis*-induced PD-L1 upregulation is dependent on *C. trachomatis* development. (**A–C**) MEFs were infected with *C. trachomatis* serovar L2 at MOI = 1 and analyzed at 6, 18, or 24 hpi for (**A**) *pdl1* expression via RT-qPCR or (**B, C**) PD-L1 surface expression via flow cytometry gated on live cells shown as (**B**) geometric mean fluorescent intensity (gMFI) or (**C**) representative histogram plots. (**D, E**) MEFs were infected with *C. trachomatis* serovar L2 at MOI = 1 and analyzed at 24 hpi for (**D**) *pdl1* expression via RT-qPCR or (**E**) PD-L1 surface expression via flow cytometry gated on live cells shown as gMFI. Inoculum media were replaced with media containing C1 or DMSO at the indicated time points. All mock infections were performed using SPG buffer. All data shown are representative of at least two independent experiments. According to data normality, data were analyzed using (**A, B**) an unpaired *t*-test, (**D**) Kruskal–Wallis test, or (**E**) one-way analysis of variance (ANOVA). Error bars = SEM, ns = non-significant, **P < 0.01, ***P < 0.001, ****P < 0.0001.

*C. trachomatis* has been shown to upregulate PD-L1 *in vitro* at 18 hpi via flow cytometry (8). We sought to expand on this finding by performing a time course of infection to pinpoint peak PD-L1 expression at the transcript and protein surface expression level via RT-qPCR and flow cytometry, respectively. At 6 hpi, there is modest upregulation of *pdl1* transcription, but no surface expression of PD-L1 (Fig. 1A through C). Robust *pdl1* mRNA upregulation occurs at 18 hpi and is maintained at 24 hpi. However, PD-L1 surface expression peaks at 24 hpi. Additionally, UV-inactivated *C. trachomatis* fails to induce upregulation of PD-L1 (Fig S1A and B). These data suggest that some *C. trachomatis* activity that occurs between 6 and 18 hpi triggers PD-L1 upregulation.

*C. trachomatis* uses its T3SS to secrete effector proteins into host cells at various stages of its lifecycle (24, 25). Thus, we wanted to assess if PD-L1 upregulation is dependent on the *C. trachomatis* T3SS. To address this, we used N′-(3,5-dibromo-2-hydroxybenzylidene)-4-nitrobenzohydrazide (C1), which has been shown to inhibit the *Chlamydia* T3SS and development cycle (26–28). We added C1 at 30 min, 6 h, and 12 h post-infection at various concentrations to inhibit the T3SS and *Chlamydia* development at different stages. Importantly, we observed minimal to no difference in the percentage of live MEFs following C1 addition, indicating that C1 is not toxic to MEFs (Fig S2). Interestingly, PD-L1 expression is significantly reduced when adding C1 at all time points (Fig. 1D and E), although reduction in PD-L1 surface expression is most significant at earlier time points of C1 addition. This suggests that PD-L1 upregulation requires *C. trachomatis* development via its T3SS, or alternatively, an effector secreted by the T3SS or T3SS itself may be responsible for upregulating PD-L1.

### *C. trachomatis* induces a heat-labile secreted factor that is sufficient to upregulate PD-L1 *in vitro*

**Fig 2:**
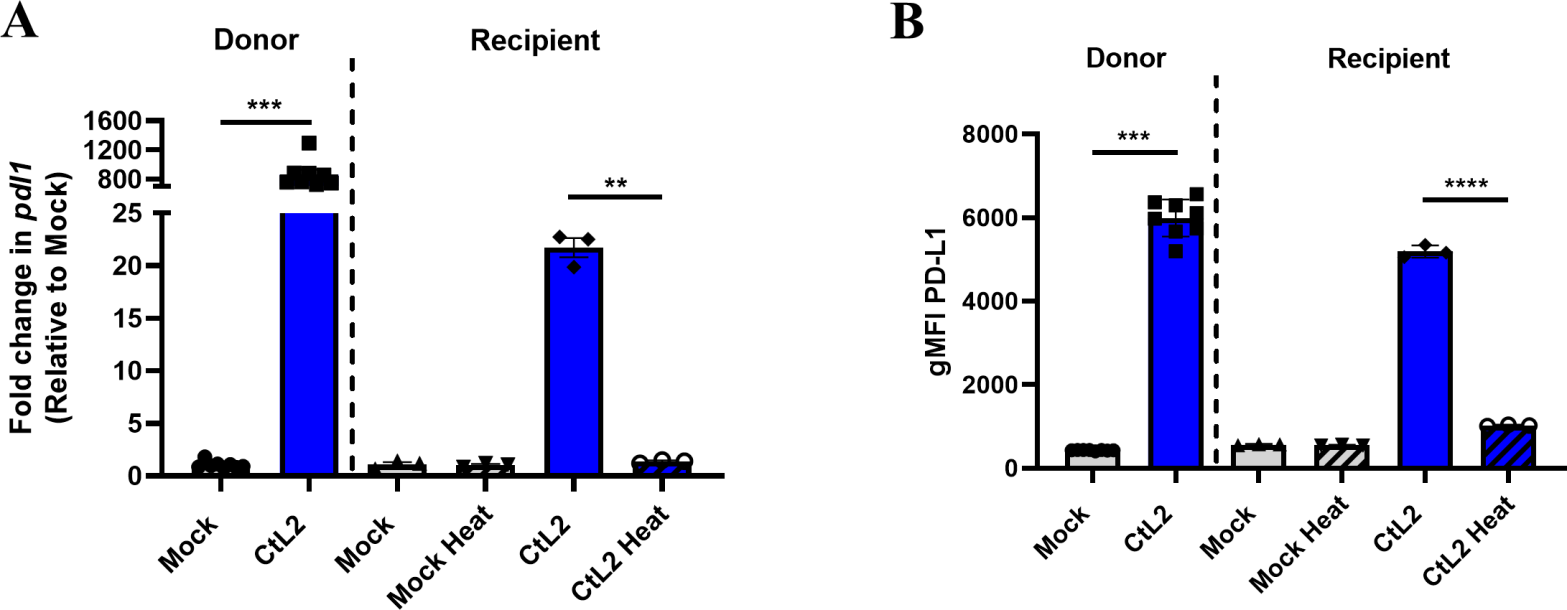
*C. trachomatis* induces a heat-labile secreted factor that is sufficient to upregulate PD-L1 *in vitro*. (**A, B**) MEFs were infected with *C. trachomatis* serovar L2 at MOI = 1 (donor), and at 24 hpi, supernatants were collected, filter sterilized, and transferred to naïve, uninfected MEFs (recipient) for 24 h. Where indicated, supernatants from donor MEFs were heated at 56°C for 30 min prior to transfer onto naïve MEFs. Donor and recipient MEFs were analyzed for (**A**) *pdl1* expression via RT-qPCR or (**B**) PD-L1 surface expression via flow cytometry gated on live cells shown as gMFI at 24 hpi (donor) and 24 h post supernatant transfer (recipient). All mock infections were performed using SPG buffer. All data shown are representative of at least two independent experiments. According to data normality, data were analyzed using Mann–Whitney U *t*-test (A, donor; B, donor), unpaired *t*-test (A, recipient), or Welch’s *t*-test (B, recipient). Error bars = SEM, **P < 0.01, ***P < 0.001, ****P < 0.0001.

We next examined whether PD-L1 upregulation occurred only in directly infected cells or if infected cells could secrete a factor that can induce PD-L1 upregulation on non-infected cells. To answer this, we collected filter-sterilized supernatant from infected MEFs (donor) 24 hpi and transferred this supernatant onto naïve uninfected MEFs (recipient) for 24 h. Recipient MEFs significantly upregulated PD-L1 at the transcript (Fig. 2A) and protein surface expression level (Fig. 2B). This indicates that a secreted factor produced during *C. trachomatis* infection is sufficient to upregulate PD-L1 on uninfected cells. Moreover, this secreted factor is heat labile, since heating the donor supernatant at 56°C for 30 min abrogates upregulation of PD-L1 (Fig. 2C and D).

**Fig 3:**
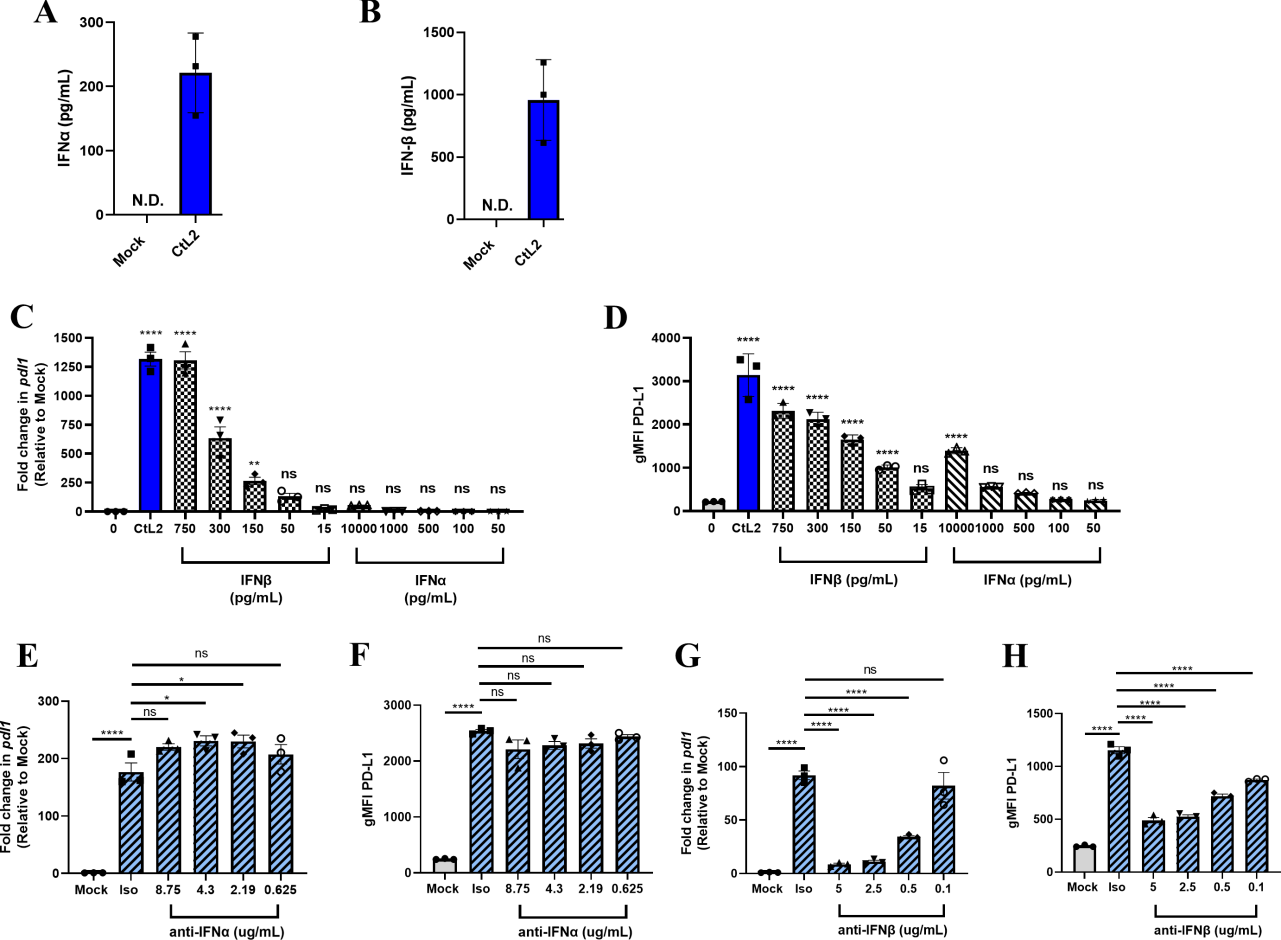
IFN-β contributes to PD-L1 upregulation during *C. trachomatis* infection *in vitro*. (**A, B**) At 24-h post *C. trachomatis* serovar L2 MOI = 1 infection, MEF supernatants were analyzed for the presence of (**A**) IFN-α or (**B**) IFN-β via ELISA. (**C, D**) MEFs were incubated with filter-sterilized supernatant from *C. trachomatis* serovar L2 MOI = 1 infected MEFs collected at 24 hpi (blue) or indicated concentrations of recombinant IFN-α or IFN-β for 6 h. (**E, F**) Filter-sterilized supernatant from *C. trachomatis* serovar L2 MOI = 1 infected MEFs was collected at 24 hpi, diluted (**E, F**) two- or (**G, H**) 32-fold, and subsequently incubated with isotype control antibodies, (**E, F**) anti-IFN-α, or (**G, H**) anti-IFN-β for 1 h prior to transfer onto naïve MEFs. Six hours post-incubation, (**C, E, G**) *pdl1* expression was measured by RT-qPCR, and (**D, F, H**) PD-L1 surface expression was measured by flow cytometry gated on live cells shown as gMFI. All mock infections were performed using SPG buffer. All data shown are representative of at least two independent experiments. Data normality was confirmed and analyzed using ANOVA. N.D., not detected; error bars = SEM; ns, non-significant; *P < 0.05, **P < 0.01, ****P < 0.0001.

**Fig 4:**
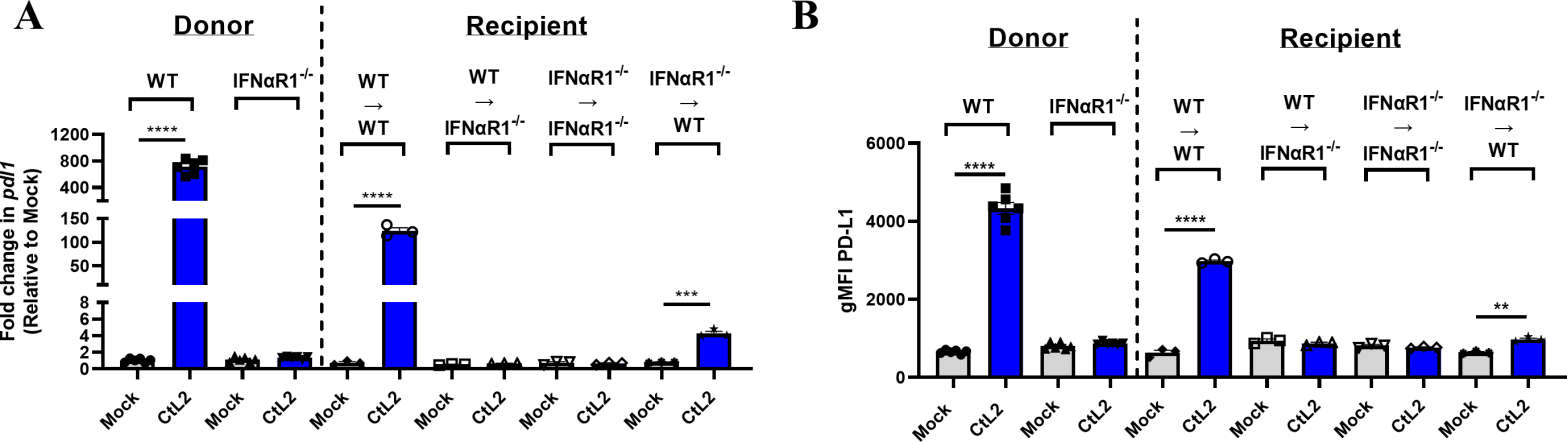
Type I IFN sensing is required for PD-L1 upregulation during *C. trachomatis* infection *in vitro*. (**A, B**) WT or IFNαR1^−/−^ MEFs were infected with *C. trachomatis* serovar L2 at MOI = 1 (donor), and at 24 hpi, supernatants were collected, filter sterilized, and transferred to naïve, uninfected WT or IFNαR1^−/−^ MEFs (recipient). Donor and recipient MEFs were analyzed for (**A**) *pdl1* expression via RT-qPCR or (**B**) PD-L1 surface expression via flow cytometry gated on live cells shown as gMFI at 24 hpi (donor) and 6 h post supernatant transfer (recipient). All mock infections were performed using SPG buffer. All data shown are representative of at least two independent experiments. Data normality was confirmed and analyzed using an unpaired *t*-test. Error bars = SEM, **P < 0.01, ***P < 0.001, ****P < 0.0001.

Interestingly, recipient MEFs upregulate *pdl1* mRNA at lower levels than donor MEFs at 24 h post supernatant transfer despite similar PD-L1 surface expression levels (Fig. 2A and B). This suggests that the secreted factor that induces PD-L1 expression may induce *pdl1* transcription more rapidly than at 24 h post supernatant transfer, and *pdl1* transcripts may wane while PD-L1 surface expression is maintained. This is supported by Fig. 3C and 4A; Fig S3A, which show higher levels of *pdl1* transcription post 6-h supernatant transfer.

### IFN-β contributes to upregulation of PD-L1 during *C. trachomatis* infection *in vitro*

Given that a secreted factor sufficient to upregulate PD-L1 is heat labile, we considered the possibility that this factor was a protein(s). One such candidate protein family is the type I interferons (IFNs), which, in humans, include 13 subtypes of IFN-α and single types each of IFN-β, IFN-ω, IFN-κ, and IFN-ε (29). Previous work has shown that both IFN-α and IFN-β can induce PD-L1 expression *in vitro* (30). Thus, we wanted to determine if type I IFNs contribute to PD-L1 upregulation during *C. trachomatis* infection.

A previous work has shown that type I IFNs are robustly induced during *C. trachomatis* infection (31–33). In line with these previous findings, we observed robust secretion of IFN-β (Fig. 3B) and, to a lesser extent, IFN-α following 24 h of *C. trachomatis* infection in MEFs (Fig. 3A). To test if these observed concentrations of IFN-α and IFN-β are sufficient to upregulate PD-L1 to the same extent as the filter-sterilized supernatant from *C. trachomatis*-infected MEFs, we performed a titration of recombinant IFN-α and IFN-β on uninfected MEFs. The same amount of IFN-β observed in Fig. 3B is sufficient to upregulate PD-L1 to the same extent as filter-sterilized supernatant from infected MEFs (Fig. 3C and D). In contrast, a 100-fold higher concentration of observed IFN-α during *C. trachomatis* infection was required for moderate PD-L1 upregulation (Fig. 3C and D). Therefore, we hypothesized that IFN-β, but not IFN-α, contributes to the upregulation of PD-L1 during *in vitro* infection of MEFs. To confirm our hypothesis, we performed a neutralizing antibody experiment using anti-IFN-α and anti-IFN-β antibodies. Due to the lower efficacy of the neutralizing antibodies and the high levels of observed IFN-α and IFN-β secretion (Fig. 3A and B), we diluted the filter-sterilized supernatant from infected MEFs twofold for anti-IFN-α experiments (Fig. 3E and F) and 32-fold for anti-IFN-β experiments (Fig. 3G and H). We confirmed that PD-L1 upregulation is still detectable even at 32-fold dilution (Fig S3A and B), which underscores the potency of the filter-sterilized supernatant from infected MEFs in its ability to induce upregulation of PD-L1. Only anti-IFN-β and not anti-IFN-α neutralizing antibodies were able to significantly decrease PD-L1 upregulation (Fig. 3E through H). Taken together, these data show that IFN-β, but not IFN-α, significantly contributes to upregulation of PD-L1 on MEFs *in vitro*.

### Type I IFN sensing is necessary for upregulation of PD-L1 during *C. trachomatis* infection *in vitro*

We next sought to assess if type I IFNs are required for upregulation of PD-L1 *in vitro*. All type I IFNs signal through IFNαR, which is composed of IFNαR1 and IFNαR2 subunits (29). Thus, we created IFNαR1^−/−^ MEFs, which are incapable of sensing any type I IFNs. We first determined that *C. trachomatis* growth and development are comparable in WT and IFNαR1^−/−^ MEFs, showing that there is no significant difference in inclusion forming unit (IFU) production between WT and IFNαR1^−/−^ MEFs (Fig S4A). Interestingly, complete abrogation of *C. trachomatis*-induced PD-L1 upregulation was observed in IFNαR1^−/−^ MEFs (Fig. 4A and B, donor). The filter-sterilized supernatant from WT MEFs upregulated PD-L1 on WT but not IFNαR1^−/−^ MEFs (Fig S4B and C recipient). In contrast, the filter-sterilized supernatant from IFNαR1^−/−^ MEFs failed to induce upregulation of PD-L1 on IFNαR1^−^/^−^ MEFs and induced modest PD-L1 upregulation on WT MEFs. However, this failure is due to a lack of type I IFN secretion (Fig S4B and C) likely driven by the loss of autocrine/paracrine signaling through IFNαR1, which has been previously described (34, 35). These data indicate that sensing of type I IFNs is required for upregulation of PD-L1 during *C. trachomatis* infection *in vitro*.

### Type I IFNs contribute to upregulation of PD-L1 *in vivo*

**Fig 5:**
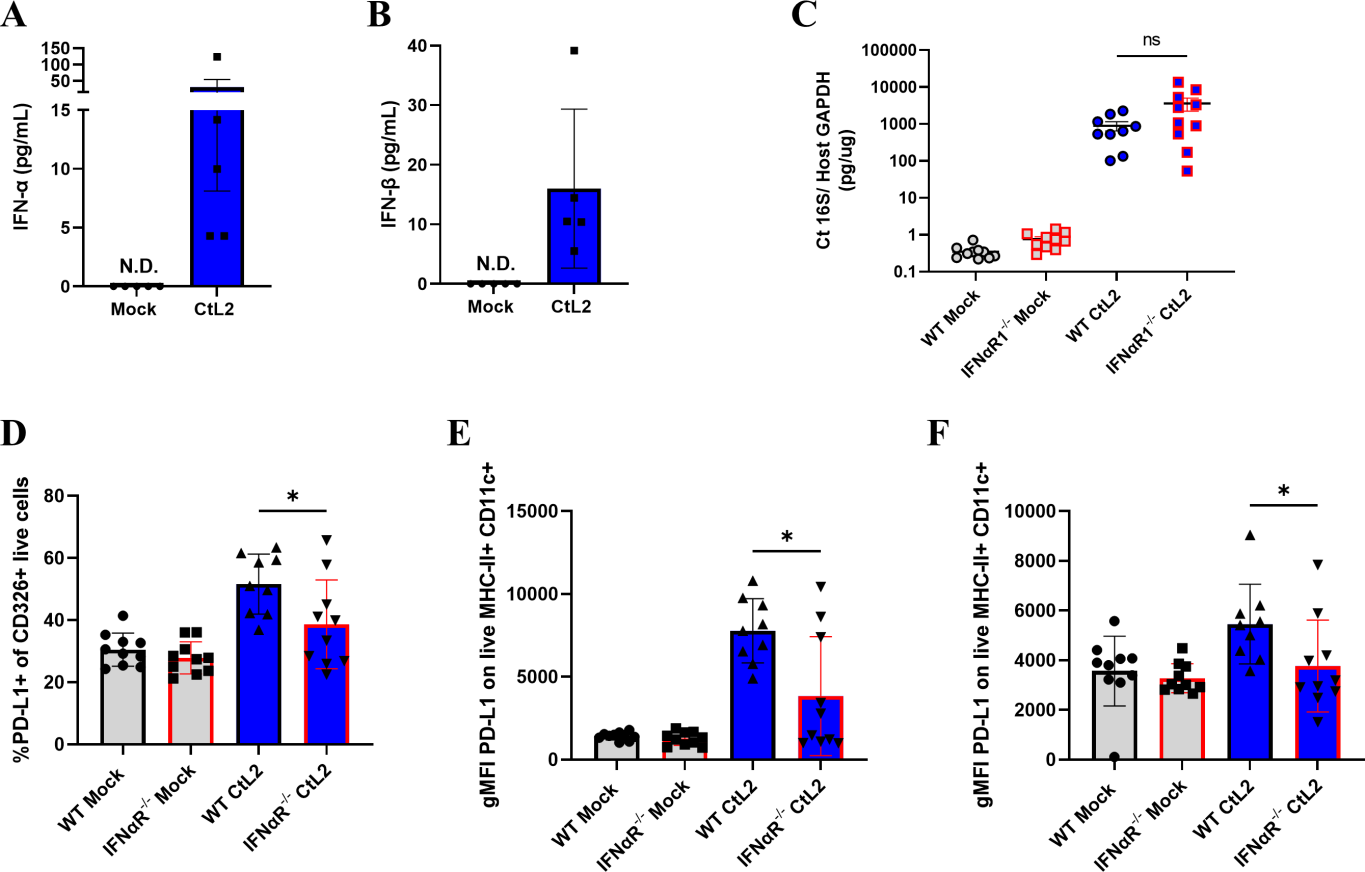
Type I IFNs contribute to PD-L1 upregulation during *C. trachomatis* infection *in vivo*. Mice were transcervically infected with 5 × 10^6^ IFUs of *C. trachomatis* serovar L2. At 24 hpi, mice were sacrificed, and the upper genital tracts and uterine draining lymph nodes were harvested. (A) IFN-α and (B) IFN-β levels in the uterus were measured via ELISA. (C) Bacterial burden in the uterus was analyzed by qPCR. PD-L1 surface expression was measured by flow cytometry on gated live (D) CD326+ epithelial cells, (E) MHC-II+Cd11c+ uterine dendritic cells (DCs), or (F) MHC-II+Cd11c+ DCs in the uterine draining lymph nodes shown as geometric mean fluorescent intensity (gMFI) or %PD-L1+. All mock infections were performed using sucrose-phosphate-glutamate (SPG) buffer. Data are (A, B) representative of at least 2 independent experiments or (C–F) pooled from 2 independent experiments. According to data normality, data were analyzed using (C, E, F) Mann-Whitney U test or (D) an unpaired t test. Error bars = SEM, ns= non-significant, * *P*< 0.05.

To validate that type I IFNs are secreted *in vivo*, we transcervically infected mice with *C. trachomatis* and assessed IFN-α and IFN-β secretion via ELISA. Both IFN-α and IFN-β were detected in the uterus of *C. trachomatis*-infected mice (Fig. 5A and B) but not in the uterine draining lymph nodes (unpublished observations). To test if type I IFNs contribute to upregulation of PD-L1 *in vivo*, we transcervically infected WT or IFNαR1^−/−^ mice with *C. trachomatis* and assessed PD-L1 expression via flow cytometry. Previous work has shown that *in vivo C. trachomatis* infection induces upregulation of PD-L1 on uterine epithelial cells and on dendritic cells (DCs) in the uterine draining lymph nodes (8). Despite similar burden in WT and IFNαR1^−/−^ mice (Fig. 5C), we observed significantly less PD-L1 expression on uterine epithelial cells (Fig. 5D; Fig S5), uterine DCs (Fig. 5E; Fig S5), and DCs in the uterine draining lymph nodes (Fig. 5F; Fig. S6) in IFNαR1^−/−^ mice compared to WT mice. This confirms that type I IFNs contribute to PD-L1 upregulation during early *in vivo* infection

## DISCUSSION

Upregulation of the immunoinhibitory ligand PD-L1 has previously been shown to contribute to the defective CD8^+^ T-cell response observed during *C. trachomatis* infection (8). However, the underlying *Chlamydia*-induced host pathways and conditions that occur early during infection that may negatively influence CD8^+^ T-cell priming and lead to PD-L1 upregulation had yet to be explored. In this study, we discovered that *C. trachomatis* infection causes secretion of type I IFNs by the host that contribute to PD-L1 upregulation during infection.

For some years now, it has been well-established that certain types of cancers are capable of upregulating PD-L1 and other costimulatory receptors and ligands to evade T-cell immunity (36). In fact, checkpoint blockade immunotherapy against PD-L1 or other co-inhibitory pathways can enhance CD8^+^ T cell-mediated killing of cancer cells, which can significantly improve tumor regression in some patients (37). Interestingly, the PD-1/PD-L1 pathway has also been shown to be exploited by *C. trachomatis* to evade CD8^+^ T-cell immunity (8). Blocking this pathway results in faster clearance of *C. trachomatis* and enhanced CD8^+^ T cell-mediated protection from infection. Thus, manipulation of costimulatory pathways seems to be a shared and effective mechanism by which cancer cells or pathogens, such as *C. trachomatis*, chronically persist within the host.

In this study, we demonstrated that between 6 and 18 hpi, *C. trachomatis* begins to induce robust upregulation of PD-L1. We also established that PD-L1 upregulation is dependent on the T3SS and *Chlamydia* development using the T3SS inhibitor C1 and UV inactivation (Fig. 1D and E; Fig S1A and B). Additionally, we determined that *C. trachomatis* induces type I IFN secretion (Fig. 3A, B and 5A, B) and that sensing of type I IFNs is required for PD-L1 upregulation *in vitro* (Fig. 4) and contributes to upregulation of PD-L1 *in vivo* (Fig. 5D through F). Interestingly, previous work has shown that interferon-stimulated genes (ISGs) are transcriptionally upregulated around 18 hpi but not 8 hpi during *Chlamydia pneumoniae* infection in murine fibroblasts (38). Furthermore, upregulation of ISGs is dependent on the T3SS and *Chlamydia* development since C1 addition and UV inactivation of *C. pneumoniae* abrogates upregulation of ISGs (38). These data are in line with our observations and provide further support that T3SS-dependent induction of type I IFNs contributes to upregulation of PD-L1.

Here, we have established that IFN-β is the dominant factor responsible for upregulating PD-L1 *in vitro* in MEFs (Fig. 3). To determine if IFN-β is responsible for type I IFN-driven upregulation of PD-L1 *in vivo*, we performed a neutralizing antibody experiment in mice. Although we observed a reduction in PD-L1 expression in anti-IFNαR1-treated mice, there was no observed reduction in PD-L1 expression in anti-IFN-β-treated mice (unpublished observations). This suggests that multiple type I IFNs could be required for type I IFN-driven upregulation of PD-L1, or alternatively, a type I IFN other than IFN-β is responsible. Given that *in vivo* infection results in slightly more IFN-α than IFN-β production in the uterus (Fig. 5A and B) in contrast to the dominance of IFN-β production during *in vitro* infection of MEFs (Fig. 3A and B), it is possible that some subtypes of the type I IFN family may play an important role *in vivo*, which may not be captured during infection of MEFs. That is, different cell types may produce and respond to subtypes of the type I IFN family differentially *in vivo*. Although there is one type of IFN-β in mice, there exist 14 subtypes and three pseudogenes of murine IFN-α (39), which complicates further neutralizing antibody studies *in vivo*. To definitely determine which subtype(s) of the type I IFN family contribute to PD-L1 upregulation *in vivo*, follow-up studies are required.

In this study, we have shown that type I IFN sensing contributes to *in vivo* upregulation of PD-L1 at 24 hpi (Fig. 5). However, there is clearly another factor involved in upregulating PD-L1 during *C. trachomatis* infection. IFN-γ, a type II IFN, is a strong inducer of PD-L1 expression (30) and is highly expressed during *Chlamydia* infection (13, 40, 41). Indeed, we have observed decreased PD-L1 expression in IFNγR^−/−^ mice compared to that in WT mice at 5 days post *C. trachomatis* infection despite similar burden (unpublished observations). Notably, IFNγR^−/−^ mice still upregulate PD-L1 compared to mock-infected mice. Moreover, mice treated with anti-IFNγ and anti-IL-12 antibodies at 4 days post *C. trachomatis* infection exhibit reduced PD-L1 expression on uterine epithelial cells and splenic DCs at 7 dpi (10). We are currently working to determine the extent to which type I IFNs and IFN-γ may synergistically upregulate PD-L1 *in vivo*. This hypothesis could also explain why we were unable to observe a difference in PD-L1 expression at 5 dpi between WT and IFNαR1^−/−^ mice (unpublished observations), especially given that IFN-γ production has been observed to peak between 2 and 5 days post-infection (42, 43). That is, IFN-γ-induced PD-L1 upregulation at 5 dpi may mask any effect type I IFNs may have on PD-L1 expression at this later time point.

Interestingly, type I IFNs have been implicated in hindering T-cell responses during *Chlamydia* infection in other work where IFNαR1^−/−^ mice (42) or knockouts of the IFN-β transcriptional regulator IRF3 in mice (44) both result in enhanced T-cell responses following *C. muridarum* infection. Type I IFNs stimulated during acute infection also can be beneficial for enhancing T-cell responses via enhanced DC activation and function (45). However, chronic infections resulting in sustained type I IFN expression that correlate with increased immunosuppressive signaling, including PD-1/PD-L1, have been shown to negatively impact T-cell function and pathogen clearance (46–48). Interestingly, combination type I IFN treatment with PD-1/PD-L1 blockade has been shown to potentially boost T-cell responses to certain cancers by increasing antigen presentation, controlling proliferation, and sensitizing tumor cells to PD-1/PD-L1 blockade (49). However, responses to this combination treatment may be heterogenous as one report indicates that type I IFN may lead to PD-1/PD-L1 blockade resistance through the induction of nitric oxide synthase 2 (NOS2) expression in tumor cells and DCs leading to T-cell dysfunction and an upregulation of Tregs (50). Given our findings that type I IFNs contribute to the upregulation of PD-L1, it is intriguing to hypothesize that this pathway contributes to the suboptimal T-cell responses observed in previous studies (42, 44) and how it may contribute to chronic, persistent infection in the host. However, apart from contributing to PD-L1 upregulation, type I IFNs may play an important role in coordinating adaptive immunity by boosting APC responses that should be explored in future studies.

A previous work has shown that PD-L1^−/−^ mice contain significantly more CD8^+^ T cells specific to the *C. trachomatis* antigen CrpA in the uterus following secondary infection compared to WT mice (8). To determine if type I IFNs contribute to an impaired secondary CD8^+^ T-cell response, we tracked CrpA^+^ CD8^+^ T cells in WT and IFNαR1^−/−^ mice at 5 days post-secondary infection. Although data suggest that IFNαR1^−/−^ mice exhibit reduced PD-1 expression on CrpA^+^ and CrpA^−^ CD8^+^ T cells in the uterus, IFNαR1^−/−^ mice seem to exhibit the same number of CrpA^+^ CD8^+^ T cells in the uterus compared to WT mice (unpublished observations). This suggests that, although type I IFNs appear sufficient to induce PD-1 expression on CD8^+^ T cells, they alone may not be sufficient to impair CD8^+^ T-cell function. Given that *in vivo* PD-L1 upregulation during *C. trachomatis* infection is multi-factorial and may involve IFN-γ, it is possible that multiple factors are required to observe an impaired CD8^+^ T-cell response similar to that observed in PD-L1^−/−^ mice (8).

Despite the public health burden caused by *C. trachomatis* infection, an effective vaccine has yet to be developed (51). Natural immunity has previously been used to guide effective vaccine strategies against pathogens (51–53). However, some patients previously infected and cured of *C. trachomatis* following antibiotic treatment remain susceptible to repeat infections (6). One explanation for this failure to protect against reinfection may be that antibiotic treatment arrests the development of robust adaptive immunity. Furthermore, manipulation of immune checkpoint pathways, such as the PD-1/PD-L1 pathway by *C. trachomatis* (8), may lead to an even more ineffective natural adaptive immune response. Strategic and specific blockade of these pathways during vaccination could potentially elicit a more robust, protective T-cell response (8, 54) while minimizing pathological effects that have been previously reported with global immune checkpoint blockade during *Chlamydia* infection (55). To identify strategic points of intervention within the PD-1/PD-L1 pathway during *C. trachomatis* infection, we are investigating which *C. trachomatis* factor(s) are responsible for inducing type I IFN-driven PD-L1 upregulation. Ultimately, this knowledge may be useful in the development of vaccination approaches that redirect immune stimulation in a manner that overcomes the limitations of natural immunity.

## SUPPLEMENTAL MATERIAL

Supplemental figures iai.00040-25-s0001.pdf Fig. S1 to S6.

ASM does not own the copyrights to Supplemental Material that may be linked to, or accessed through, an article. The authors have granted ASM a non-exclusive, world-wide license to publish the Supplemental Material files. Please contact the corresponding author directly for reuse.
