# Supplementary material for: Type I IFNs contribute to upregulation of PD-L1 during Chlamydia trachomatis infection
Source: Infect Immun. 2025 Mar 12;93(4):e00040-25. doi: 10.1128/iai.00040-25 (PMC11977314; doi:10.1128/iai.00040-25)
Supplement: Supplemental figures — Fig. S1 to S6. [file iai.00040-25-s0001.pdf]

# Supplementary Figure 1

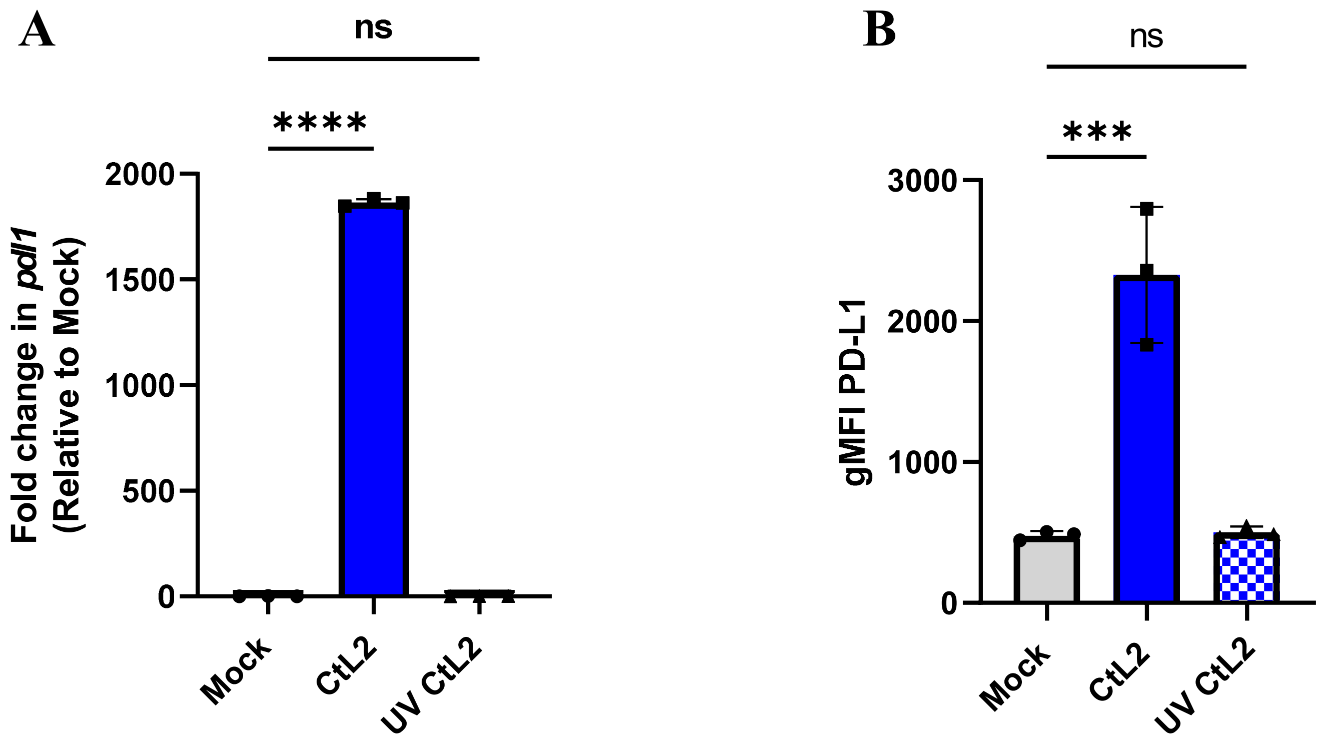


**Supplementary Figure 1. UV-inactivated *C. trachomatis* fails to induce upregulation of PD-L1 *in vitro*.** **(A-B)** MEFs were infected with live or UV-inactivated *C. trachomatis* serovar L2 at MOI=1 and analyzed at 24hpi for **(A)** *pdl1* expression via RT-qPCR or **(B)** PD-L1 surface expression via flow cytometry gated on live cells shown as geometric mean fluorescent intensity (gMFI). All data shown are representative of at least 2 independent experiments. All mock infections were performed using sucrose-phosphate-glutamate (SPG) buffer. Data normality was confirmed and analyzed using a one-way ANOVA. Error bars = SEM, ns= non-significant, *** $p< 0.001$,**** $p< 0.0001$

# Supplementary Figure 2

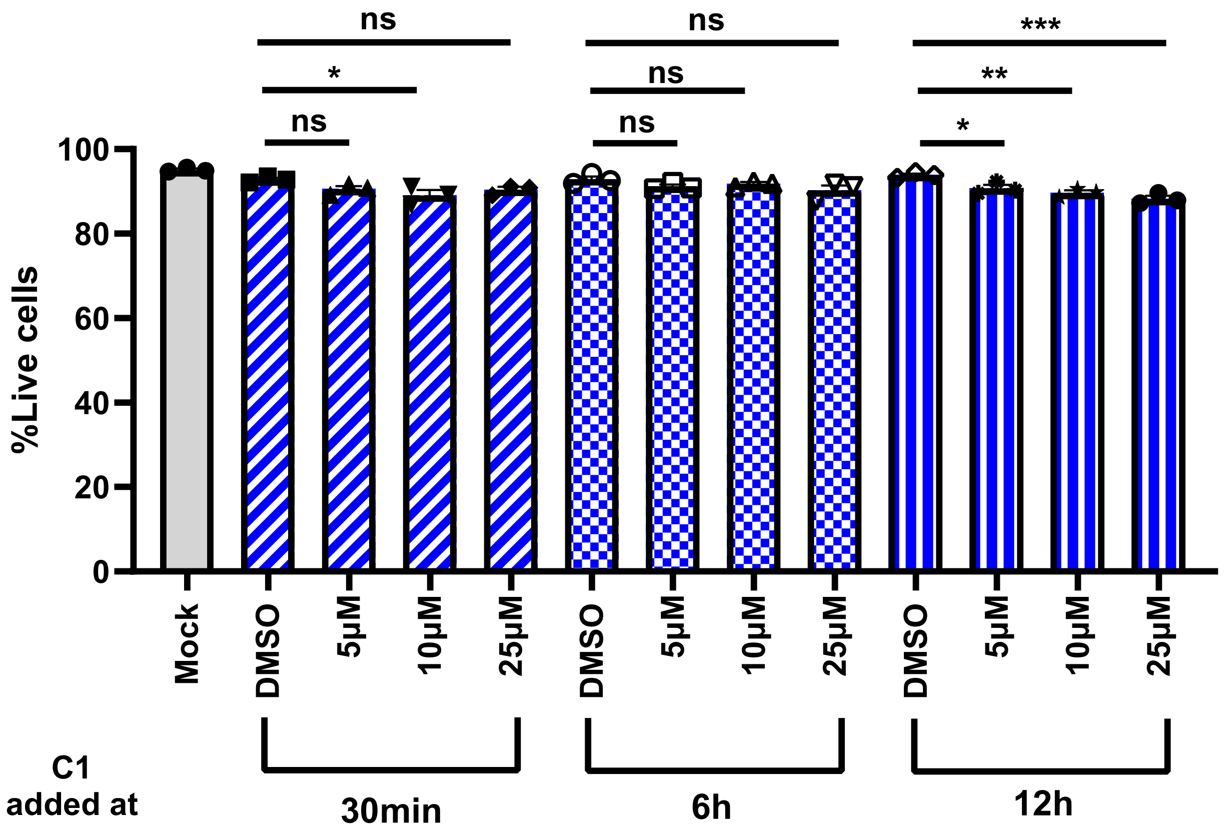


**Supplementary Figure 2. C1 is non-toxic to MEFs during *C. trachomatis* infection.** MEFs were infected with *C. trachomatis* serovar L2 at MOI=1 and analyzed at 24hpi for percentage of live cells. Inoculum media was replaced with media containing C1 or DMSO at the indicated time points. All mock infections were performed using sucrose-phosphate-glutamate (SPG) buffer. All data shown are representative of at least 2 independent experiments. According to data normality, data were analyzed using an ordinary one-way ANOVA. Error bars = SEM, ns= non-significant, *$p<0.05$, **$p<0.01$, *** $p< 0.001$.

# Supplementary Figure 3

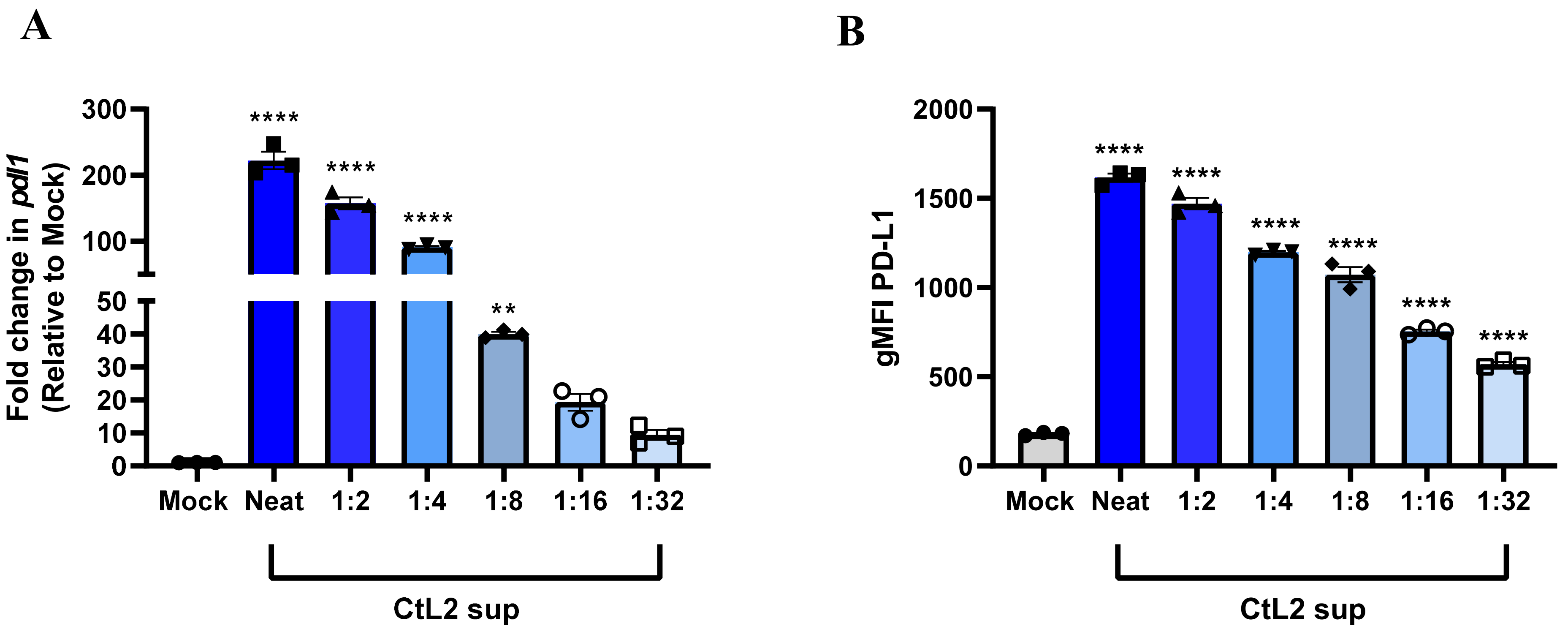


**Supplementary Figure 3. Supernatant from *C. trachomatis* infected MEFs potently induces PD-L1 upregulation. (A-B)** MEFs were incubated with filter sterilized supernatant from *C. trachomatis* serovar L2 MOI=1 infected MEFs collected at 24hpi. 6h post supernatant transfer, MEFs were analyzed for **(A)** *pdl1* expression via RT-qPCR or **(B)** PD-L1 surface expression via flow cytometry gated on live cells shown as geometric mean fluorescent intensity (gMFI). All mock infections were performed using sucrose-phosphate-glutamate (SPG) buffer. All data shown are representative of at least 2 independent experiments. Data normality was confirmed and analyzed using a one-way ANOVA. Error bars = SEM, ** $p<0.01$, **** $p< 0.0001$

# Supplementary Figure 4

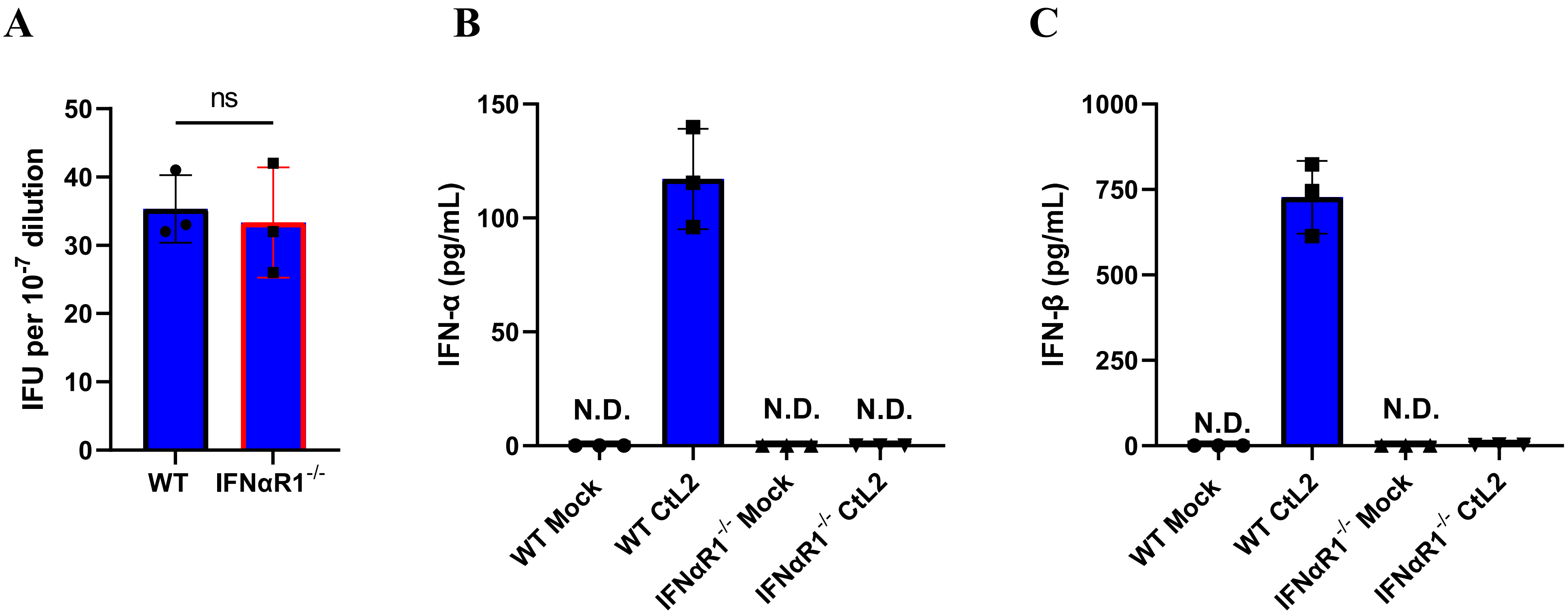


**Supplementary Figure 4. IFNαR1$^{-/-}$ MEFs fail to secrete IFN-α and IFN-β following *C. trachomatis* infection. (A)** WT or IFNαR1$^{-/-}$ MEFs were infected with *C. trachomatis* serovar L2 at a $10^{-7}$ dilution of stock *C. trachomatis*. 30hpi, IFUs were enumerated via immunofluorescence staining of inclusions. **(B-C)** 24h post *C. trachomatis* serovar L2 MOI=1 infection, MEF supernatants were analyzed for the presence of **(A)** IFNα or **(B)** IFNβ via enzyme-linked immunosorbent assay (ELISA). All mock infections were performed using sucrose-phosphate-glutamate (SPG) buffer. All data shown are representative of at least 2 independent experiments. Data normality was confirmed and analyzed using **(A)** an unpaired t test. N.D.= not detected. Error bars = SEM, ns= non-significant.

# Supplementary Figure 5

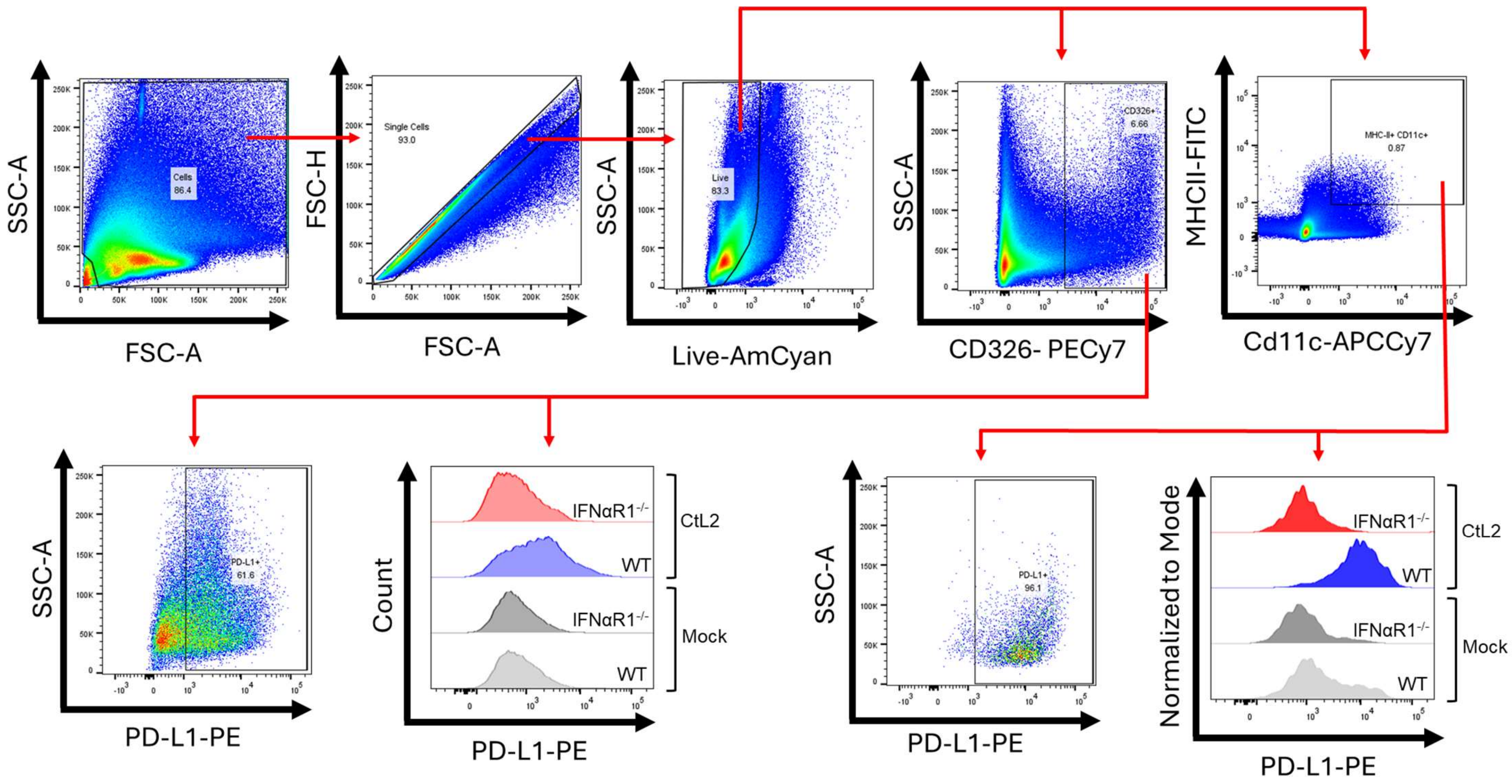


**Supplementary Figure 5. Gating strategy for PD-L1 expression analysis on uterine epithelial cells and DCs.** Representative flow cytometry plots for analysis of PD-L1 expression on gated uterine epithelial cells and uterine DCs. Representative histogram plots of PD-L1 expression are also included.

# Supplementary Figure 6

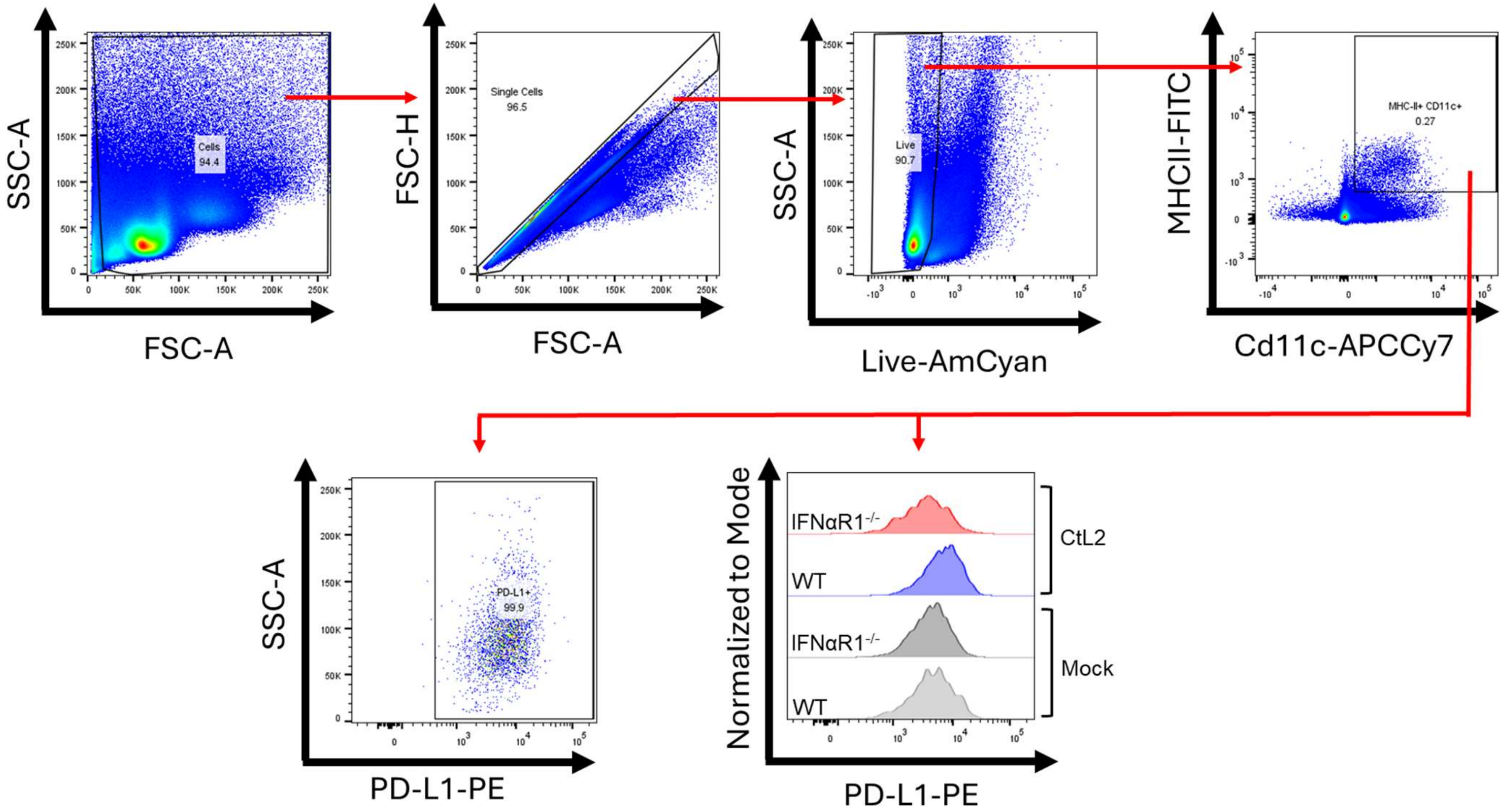


**Supplementary Figure 6. Gating strategy for PD-L1 expression analysis on uterine draining lymph node (dLN) DCs.** Representative flow cytometry plots for analysis of PD-L1 expression on gated dLN DCs. Representative histogram plots of PD-L1 expression are also included.
